# Unveiling Prognostic RNA Biomarkers through a Multi-Cohort Study in Colorectal Cancer

**DOI:** 10.3390/ijms25063317

**Published:** 2024-03-14

**Authors:** Zehwan Kim, Jaebon Lee, Ye Eun Yoon, Jae Won Yun

**Affiliations:** 1 Department of Laboratory Medicine, Yeungnam University College of Medicine, Daegu 42415, Republic of Korea; 2 Veterans Health Service Medical Research Institute, Veterans Health Service Medical Center, Seoul 05368, Republic of Korea

**Keywords:** prognostic biomarkers, colorectal cancer, gene expression, RNA

## Abstract

Because cancer is a leading cause of death and is thought to be caused by genetic errors or genomic instability in many circumstances, there have been studies exploring cancer’s genetic basis using microarray and RNA-seq methods, linking gene expression data to patient survival. This research introduces a methodological framework, combining heterogeneous gene expression data, random forest selection, and pathway analysis, alongside clinical information and Cox regression analysis, to discover prognostic biomarkers. Heterogeneous gene expression data for colorectal cancer were collected from TCGA-COAD (RNA-seq), and GSE17536 and GSE39582 (microarray), and were integrated with Entrez Gene IDs. Using Cox regression analysis and random forest, genes with consistent hazard ratios and significantly affecting patient survivability were chosen. Predictive accuracy was evaluated using ROC curves. Pathway analysis identified potential RNA biomarkers. The authors identified 28 RNA biomarkers. Pathway analysis revealed enrichment in cancer-related pathways, notably EGFR downstream signaling and IGF1R signaling. Three RNA biomarkers (ZEB1-AS1, PI4K2A, and ITGB8-AS1) and two clinical biomarkers (stage and age) were chosen for a prognostic model, improving predictive performance compared to using clinical biomarkers alone. Despite biomarker identification challenges, this study underscores integration of heterogenous gene expression data for discovery.

## 1. Introduction

Cancer is a leading cause of death [1] and colorectal cancer is the third deadliest cancer according to the epidemiology of colorectal cancer (WHO) and the second leading cause in the United States [2,3,4]. For the treatment of cancer, epidemiological studies, identification of causes, factors affecting prognosis, refinement of diagnosis, and selection of the most effective treatment for each diagnosed subtype of cancer are important. In many cases, cancer is thought to be caused by genetic errors or genomic instability [5]. In this regard, research has been ongoing to obtain information on which genes affect diagnosis, treatment, and prognosis, and precision medicine using genomic information is playing an important role [4,6,7,8].

An important part of precision medicine using genomic information is the study of gene expression. Experimental methods to check gene expression in tissues obtained from colorectal cancer have been developed, and methods with relatively low cost and effort have been developed. Among the various methods, microarray technology [9,10,11,12,13,14,15,16] or next generation sequencing (NGS)-based RNA-Seq [17,18,19,20] are the main examples.

Microarray technology, pioneered by companies such as Affymetrix and Illumina, is vital for studying mRNA abundance and gene expression. The technology employs numerous probes on a microarray platform to identify genes with significant mRNA production, forming probe sets with multiple designed probes per gene. Utilized extensively in experiments, microarray data are well-supported by robust statistical methods and established protocols. The majority of microarray data from reported studies are publicly accessible, systematically archived in databases like Gene Express Omnibus (GEO) [21] and ArrayExpress [22,23].

The RNA-seq method uses NGS technology to measure the amount of mRNA. Using the mRNA produced by the cells of a tissue of interest, complementary DNA is transcribed and the sequence of the complementary DNA can be sequenced to indirectly determine which genes are producing the mRNA. The Cancer Genome Atlas (TCGA) is a large-scale project that collects patient information as well as tissues from many types of cancer disease [24]. It also provides publicly available experimental data in terms of various aspects, such as copy number, DNA methylation, and gene expression RNA-seq.

**Figure 1:**
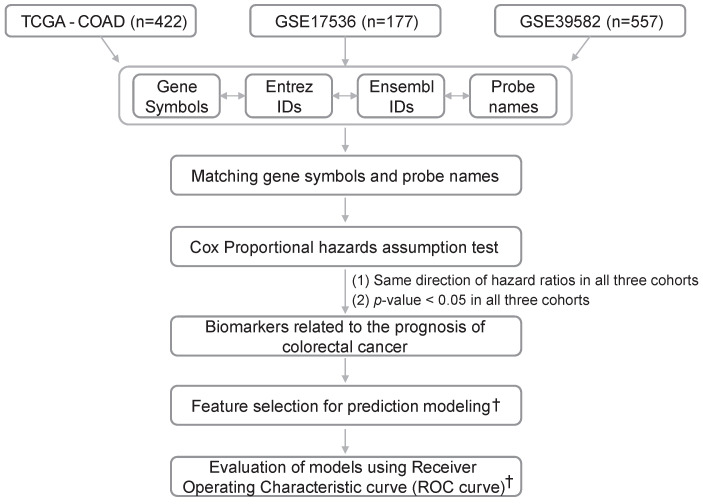
Flow chart of this study. A total of three cohorts were selected for the further analysis of finding biomarkers related to the prognosis of colorectal cancer and modeling the prediction of survival events. †: Details are described in Section 2.4.

In this study, we developed a systematic approach to discover prognostic biomarkers using a multi-cohort study and an artificial intelligence method. The aims of our study are, first, to establish a methodology to systematically identify prognostic biomarkers using multiple multi-cohort data with minimal false positives (Figure 1). The second is to use this methodology to systematically identify and describe RNA biomarkers that reflect prognosis independently of clinical parameters such as age and stage in colorectal cancer.

## 2. Results

### 2.1. Characteristics of Each Cohort

Patient characteristics and pathologic information of the three cohorts are summarized in Table 1. The mean age of patients from all three cohorts was similar, i.e., 66.4, 65.5, and 66.8 years, respectively. Regarding the gender distribution, all cohorts showed a similar proportion of female patients of around 45%. In terms of tumor stage, lower stages (stages I and II) exhibited comparable proportions of 56.4%, 45.8%, and 52.6%, respectively. Pathologic information was available only in TCGA data. According to the TCGA cohort, the most frequent histologic type was adenocarcinoma, NOS, followed by mucinous adenocarcinoma. These comprised 356 (84.4%) and 61 (14.5%) of the 422 specimens, respectively.

### 2.2. Cox Regression Analysis Test Result

Utilizing a multiple Cox regression model that accounted for factors such as age, sex, and tumor stage, 28 RNA biomarkers were identified by filtering for those with significant *p*-values and consistent hazard ratio direction (HR > 1 in all cohorts or HR < 1 in all cohorts) across the three cohorts (Table 2).

Next, pathway analysis was conducted to assess the enrichment of 28 RNA biomarkers in cancer-related pathways using CancerCompass (https://cancercompass.newgenes.org/, accessed on 6 January 2024). The top 10 cancer-related pathways included EGFR (ERBB1) downstream signaling, FGF signaling pathway, IGF1R signaling cascade, IRS-mediated signaling, and PI3K cascade (Supplementary Figure S1A,B).

Interestingly, a number of genes were found to be part of the EGFR downstream signaling pathway. *CHN2* (chimerin 2), *F2RL2* (coagulation factor II thrombin receptor like 2), and *PDPK1* (3-phosphoinositide dependent protein kinase 1) were genes in the EGFR downstream signaling pathway. The association of the genes with cancer was analyzed through a literature review and gene ontology analysis. The *CHN2* gene encodes a GTP-metabolizing protein crucial for cell proliferation and migration. The *F2RL2* gene showed a significant correlation with colorectal cancer initiation and progression in a prior study [25]. *PDPK1* has been implicated in treatment resistance and cancer cell growth across several cancer types [26,27]. Additionally, multiple insulin-like growth factor-related pathways were identified, and *FGFR4* and *PDPK1* were identified as key genes involved in the IGF1R signaling cascade and IRS-mediated signaling. FGFR4 was also reported for its important role in tumor progression, oncogenesis, and the development of treatment resistance [28].

### 2.3. Identification of Interaction among Hub Genes Using Network Analysis

**Figure 2:**
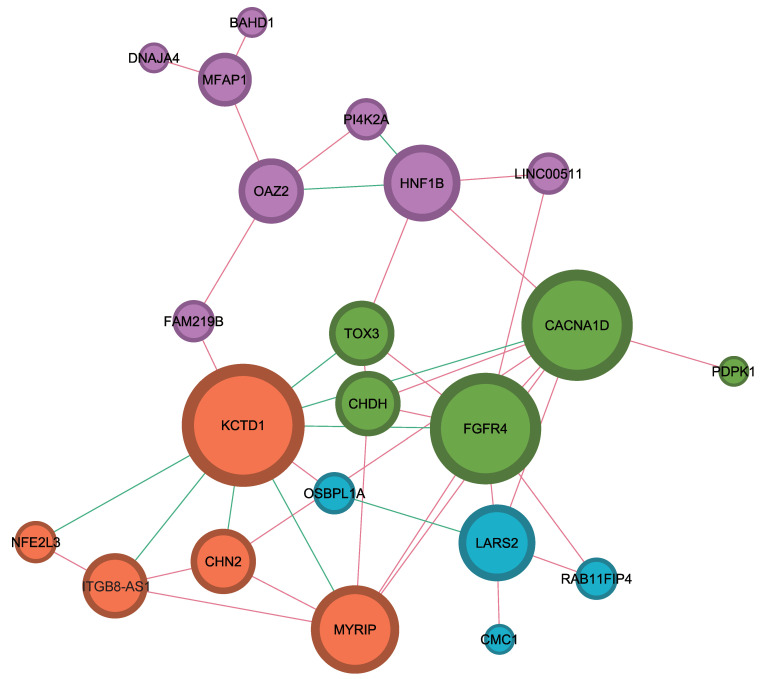
The outcome of the network analysis using the Gephi platform. The network was drawn by selecting all the relationships among the 28 genes identified using Cox regression analysis that satisfied having an absolute value of the Pearson correlation coefficient above 0.3, and a total of 22 genes were drawn. Nodes are divided into 4 classes, represented by 4 colors: purple, green, red, and cyan. The edge color in the network is orange when the correlation coefficient is higher than 0.3 and green when it is lower than −0.3.

The results from the Gephi 0.10.1 software used for network analysis of RNA biomarkers or genes are shown in Figure 2. Of the 28 genes obtained by Cox regression analysis, only 22 genes had an edge with an absolute value of the Pearson correlation R-value greater than or equal to 0.3, which were categorized into four classes. *KCTD1*, *FGFR4*, and *CACNA1D* were the most important hub genes as they were related to more than eight genes, and *HNF1B*, *LARS2*, *MYRIP*, *CHDH*, *OAZ2*, *ITGB8-AS1*, *CHN2*, and *TOX3* were also hub genes as they were related to more than four genes. *KCT1D* was highly associated with other genes in a negative correlation, and seven of the nine edges tended to be negatively co-expressed with other genes.

### 2.4. Development of Prognostic Prediction Model and Performance Evaluation Using Pivotal RNA Biomarkers

**Figure 3:**
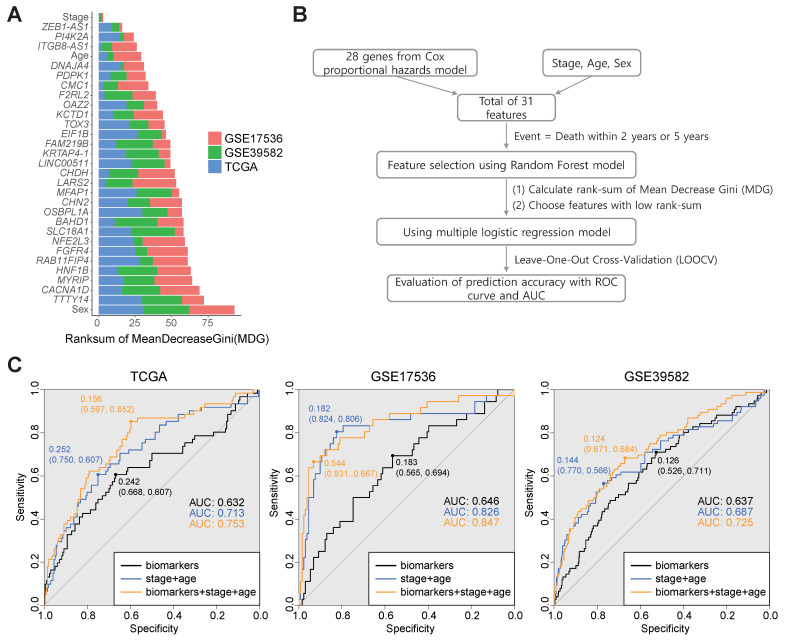
(**A**) The Ranksum of the MeanDecreaseGini (MDG) index. In each cohort, MDG for each feature was calculated using a random forest model and then ranked in descending order. After obtaining the MDG rankings in each cohort, the sum of rankings was calculated for each variable. The graph displays the sum of rankings in ascending order. (**B**) With the five features selected based on MeanDecreaseGini, a multiple logistic regression model was used to predict events such as death within 2 years. Leave-one-out cross-validation (LOOCV) was performed to evaluate prediction accuracy. (**C**) The three ROC curves were plotted based on the following criteria: (1) including only stage and age, (2) including only RNA biomarkers, and (3) including both stage, age, and RNA biomarkers. Area under the curve (AUC) values were calculated for each cohort.

To develop a prognostic prediction model for two-year survival, three pivotal RNA biomarkers were selected. In brief, MeanDecreaseGini (MDG) was computed for every biomarker in each cohort through a random forest model using the labeled information for patients based on whether they survived for more than two years or not. The MDGs for each cohort were ranked and three biomarkers from the lowest rank sum across the three cohorts were selected (Figure 3A, and details are provided in Section 4). Finally, three biomarkers, namely, ZEB1-AS1, PI4K2A, and ITGB8-AS1 (also known as CTA-293F17.1), were selected. Additionally, MDGs were calculated for three clinical biomarkers (stage, age, and gender). Among these, stage showed the lowest rank sum, while age showed the fifth lowest rank sum. In the end, three RNA biomarkers (ZEB1-AS1, PI4K2A, and ITGB8-AS1) and two clinical biomarkers (stage and age) were selected as pivotal features for the development of the model.

Next, a multiple logistic regression model for predicting two-year survival was developed using the selected five biomarkers and its performance was evaluated through ROC curve analysis (Figure 3B, and details are provided in Section 4). When using a model that included only two clinical biomarkers (stage and age) in the three cohorts, the AUC values were 0.713, 0.826, and 0.687, respectively (Figure 3C). Upon the addition of three RNA biomarkers (ZEB1-AS1, PI4K2A, and ITGB8-AS1), the AUC values improved to 0.753, 0.847, and 0.725 in the three cohorts, confirming enhancement in predictive performance.

### 2.5. The Log-Rank Test Results for the Three Biomarkers with the Highest MDG Values and the RNA Expression Heatmaps

**Figure 4:**
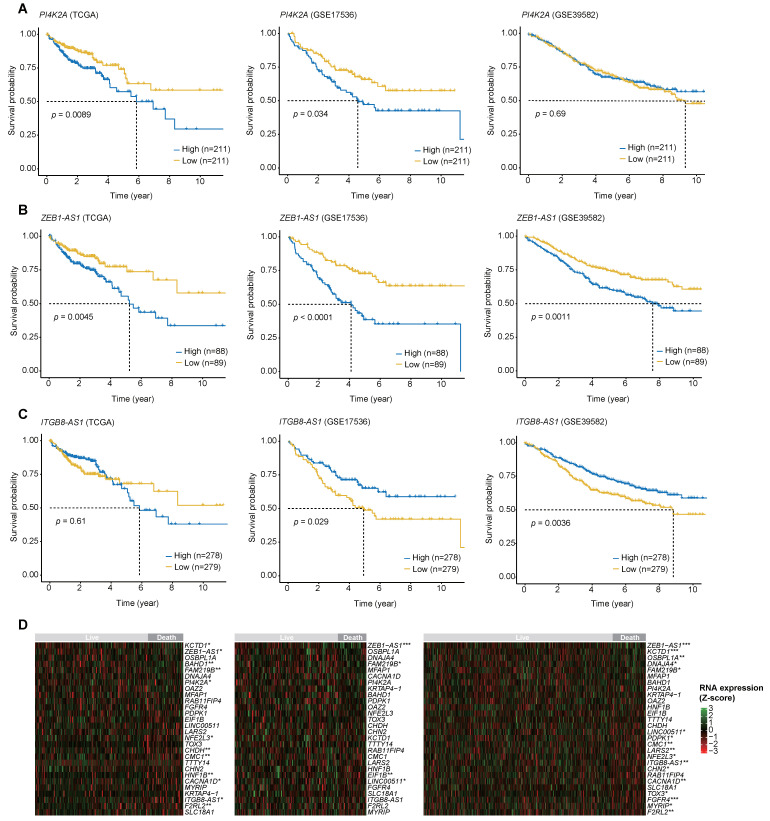
Kaplan–Meier plots of three genes and heatmaps of RNA expression levels in three cohorts. (**A**–**C**) Kaplan–Meier plot of three RNA biomarkers used in a prediction model. The high-expression and low-expression groups were distinguished using the median value of RNA expressions. (**D**) A heatmap of RNA expression levels. The RNA expression of 28 potential biomarkers selected from Cox regression analysis was transformed into Z-scores. ‘Death within 2 years’ was considered as the event. To compare RNA expression between the live group and the death group, a Wilcoxon rank-sum test was performed (***: *p*-value < 0.001, **: *p*-value < 0.01, *: *p*-value < 0.05). Left: TCGA, Middle: GSE17536, Right: GSE39582.

The results of the log-rank test performed on the three biomarkers with the highest MDG values showed that PI4K2A satisfied the *p*-value < 0.05 in two out of three cohorts, while ZEB1-AS1 satisfied the *p*-value < 0.05 in all three cohorts. For ITGB8-AS1, similar results to those of the Cox regression analysis were observed in two out of three cohorts (Figure 4A–C). The different results of the log-rank test and Cox regression analysis are discussed in the Discussion section. Next, the results of the Wilcoxon rank-sum test and RNA expression heatmaps between individuals who survived more than 2 years and those who did not showed that 12 out of 28 genes had *p*-values below 0.05 in the TCGA cohort, while 4 out of 28 genes in GSE17536, and 17 out of 28 genes in GSE39582, had *p*-values less than 0.05 (Figure 4D). ZEB1-AS1 had Wilcoxon rank-sum test *p*-values less than 0.05 in three cohorts, PI4L2A in one of three cohorts, and ITGB8-AS1 in two of three cohorts. The discrepancy between the Wilcoxon rank-sum test results and the Cox regression analysis results will also be discussed in Section 3.

## 3. Discussion

This study proposes a method for identifying prognostic RNA biomarkers while considering clinical parameters in a multi-cohort study. In total, 28 RNA biomarkers were identified, including 9 known biomarkers previously reported as prognostic factors for colorectal cancer, and 19 novel biomarkers that are, to the best of our knowledge, newly discovered (Supplementary Table S1). While some RNA biomarkers have shown associations with the carcinogenesis or metastasis of colorectal cancer, their role as prognostic biomarkers for predicting survival has not been proven. Considering consistency with previous research, the use of the proposed method with multiple well-organized cancer cohorts could enhance statistical power for discovering potential prognostic biomarkers in future studies.

The data used to measure the AUC are limited as they are categorized based on whether patients survived more than two years. Therefore, they do not provide a detailed reflection of how long each individual patient survived or the exact time of their death. Nevertheless, each biomarker underwent rigorous statistical validation in all three colorectal cancer cohorts, demonstrating independent associations with prognosis, even after accounting for stage and age. While the effect sizes of these markers may be somewhat smaller compared to well-known prognostic factors such as stage and age, which have remarkably significant effect sizes, it is still considered that these markers hold statistical significance in contributing to prognosis. In addition, while there have been recent attempts in this direction, we would assert that our work is distinct in terms of identifying prognostic biomarkers independent of stage and age [29]. The association between 28 RNA biomarkers and prognosis has been statistically evaluated using public cohorts. However, a prospective study utilizing large-scale data is needed to validate the survival effects of these biomarkers. Furthermore, there is a need to identify factors that may influence biomarkers or pathways, such as epigenetic factors or non-coding regions, in addition to RNA expression.

Conventional methods for discovering biomarkers include the log-rank test for survival analysis and statistical methods for identifying differentially expressed genes (DEGs) such as the Wilcoxon rank-sum test, DESeq2, and Limma [30,31,32]. To compare the proposed method with conventional methods, KM plots and heatmaps were generated using the three RNA biomarkers that play a pivotal role in the study. Although all three biomarkers are statistically significant in predicting prognosis with Cox regression analysis, ITGB8-AS1 and PI4K2A did not show statistical significance in one cohort each (Figure 4A–C). Cox regression analysis can be considered to have increased statistical validity by accounting for the effects of clinical information, including age, stage, and sex. On the other hand, the log-rank test has limitations for such analysis. First, without a clear biological cutoff of expression values, the challenge arises from dividing these values into two groups. Second, the log-rank test does not consider effects of other clinicopathological factors such as stage or age. In terms of the heatmap, whether the 28 potential RNA biomarkers were differentially expressed in two groups based on 2-year survival was evaluated. ZEB1-AS1 showed statistical significance in all three cohorts, whereas ITGB8-AS1 showed significance in two cohorts, and PI4K2A showed statistical significance in only one cohort, representing discrepant results compared to the results of Cox regression analysis. Like survival analysis, DEG analysis has limitations in developing prognostic biomarkers. Since case and control are selected based on survival over a certain period of time, it is difficult to perform a full evaluation that reflects the survival of individual patients. Additionally, the method for identifying DEGs does not adjust the effects of various clinical factors (age, gender, and stage). Overall, it is thought that Cox regression analysis is a favorable methodology for biomarker discovery that overcomes many of these limitations.

The EGFR downstream signaling involves the RAS/RAF/MEK/ERK, PI3K/Akt, JAK/STAT, and PLC/PKC pathways. Through these diverse downstream signaling pathways, EGFR plays an important role in the carcinogenesis of colorectal cancers [33,34]. EGFR mutation is not common in colorectal cancer patients, whereas upregulation of EGFR is common in this disease [35]. The overexpression of EGFR in colorectal cancer is associated with an advanced stage of colorectal cancer [36]. Some researchers highlighted the association between high EGFR expression level and TNM stage, especially stage T3 [37]. In mouse experiments, cells with high EGFR expression showed a higher incidence of liver metastasis [38]. However, the role of EGFR expression as a prognostic factor remains controversial [37]. EGFR also plays a pivotal role in colorectal cancer treatment. The first targeted therapeutic agent approved by the Food and Drug Administration (FDA) for colorectal cancer was cetuximab, a monoclonal antibody designed to target EGFR [39]. Furthermore, FDA approved panitumumab, another EGFR-targeting monoclonal antibody in 2006 [40]. Many studies have been conducted to identify biomarkers for selecting favorable patients for EGFR-targeted therapy, including upstream molecules, EGFR amplification, molecules involved in downstream signaling pathways, miRNAs, and methylations. However, further investigation and evaluation are still needed for clinical use of these biomarkers [35].

IGF signaling is considered to be an important factor for pathogenesis of tumors, including CRC [41]. Numerous studies revealed the correlation between IGF2 signaling and CRC [41]. IGF2 signaling accelerates cell growth and survival by activating both IGF1R and IR-A signaling [42]. In the autocrine/paracrine signaling loops of cancer cells, in particular, IGF2 working through IGF-1R and/or IR-A is frequently observed [42]. In vitro studies showed the increase in IGF2 production in diverse colon cancer cell lines [43]. In these cell lines, IGF2 overexpression was one of the key signals for the cancer cell to maintain the tumorigenic features including proliferation and differentiation [43,44]. Studies based on the publicly available data sets, including TCGA, showed that copy number changes of IGF2 and ERBB2 were observed, as well as the association between IGF2 and IGF1R with the stronger beta-catenin/TCF responsive promoter activation [41,45]. IGF2 is also one of the probable therapeutic targets of CRC, along with IGFR, ERBB2, and ERBB3 [46]. The xenografted mice were treated with IGF2R/CI-M6PR, an inhibitor of IGF2, and showed a decrease in Igf2-dependent adenoma phenotype [41,47]. In addition, commercial tissue microarray and univariate survival analysis were performed with paraffin-embedded CRC samples, and, as a result, IGF-2 expression was significantly related to a worse prognosis [48]. Moreover, since the risk of CRC development is increased with obesity and insulin resistance, the development of therapeutic technologies that target IGF signals and related proteins is warranted via further studies and clinical trials [49].

## 4. Materials and Methods

### 4.1. Data Acquisition and Preprocessing of Gene Expression Data

To obtain RNA-seq data related to colorectal cancer disease, we obtained gene expression RNA-seq data of GDC TCGA-COAD Colon Cancer (TCGA-COAD) together with clinical information (phenotype and survival data) from UCSC XENA (https://xena.ucsc.edu/, accessed on 6 January 2024) to form one cohort (n = 422) (Figure 1). For microarray data, we used two cohorts of NCBI GEO’s colon cancer from more than 100 patients, including clinical information such as age, stage, sex, and overall survival (OS) data, GSE17536, and GSE39582. We used 177 samples from GSE17536 and 557 samples from GSE39582, excluding those without age, sex, stage, or survival information (Figure 1). The microarray platforms of GSE17536 and GSE39582 were both GPL570 (Affymetrix Human Genome U133_Plus_2). Expression levels of genes were represented according to gene symbols for TCGA-COAD and probe names for GSE17536 and GSE39582 (Figure 1). Probe names were annotated with Entrez Gene IDs (Entrez IDs) and gene symbols. Probes linked to multiple gene symbols were excluded from the analysis. In cases where a single probe matched to both mRNA and miRNA, mRNA was only included in the analysis. Then, to match the gene names between cohorts, the associations between TCGA-COAD gene symbols and Ensembl gene IDs from the European Bioinformatics Institute (EBI), and Entrez IDs, were used (Figure 1).

### 4.2. Selecting a List of Genes Satisfying the Cox Proportional Hazard Assumption

Cox regression analysis was used to identify the correlation between gene expression and overall survival period. Before identifying gene expression that affects prognosis, we checked whether the proportional hazard assumption was satisfied (Figure 1). For genes that satisfied the proportional hazard assumption in a multivariate model using gene expression, age, sex, and stage, we checked the statistical significance of their hazard ratio (*p*-value less than 0.05 in all three cohorts). After assessing statistical significance, we selected a list of genes with consistent directionality of the hazard ratio (HR) in the three cohorts (HR is less than 1 or greater than 1 in all three cohorts) (Figure 1).

### 4.3. Network Analysis of Genes with Statistical Significance for Survival

We used Gephi software platform for network analysis [50]. Gephi 0.10.1 (https://gephi.org/, accessed on 6 January 2024) facilitates the analysis of interactivity interpretation of networks, leading to the identification of hub genes. Layout options include ForceAtlas 2 and overview statistics parameters include Approximate Repulsion, Dissuade Hubs, LinLog mode, Prevent Overlap, Average Degree, Average Weighted Degree, and Modularity. In the Gephi analysis, nodes were assigned gene names identified as being statistically significant using Cox regression analysis. Subsequently, we computed the average gene expression correlation R (Pearson correlation) values between genes across the three cohorts, and considered an edge to be present if the absolute value of R exceeded 0.3 for a given gene combination.

### 4.4. Feature Selection for Survival Prediction

The random forest machine learning method was employed to select features from gene expression and clinical information, including stage, age, and sex, that significantly affect the survival of patients. After events were defined as death within 2 years, survival prediction models were generated. To select features for survival models, MeanDecreaseGini (MDG) scores were calculated using the randomForest R package (https://cran.r-project.org/web/packages/randomForest/, accessed on 6 January 2024). Then, for each feature, the MDGs in each cohort were ranked and the features were prioritized based on the smallest sum of the ranks.

### 4.5. Evaluation of Predicting Accuracy

In addition to selecting features for survival prediction, receiver operating characteristic (ROC) curve analysis was conducted to assess whether the selected biomarkers could enhance survival prediction. The multiple logistic regression method was used as the prediction model. Using the model, the leave-one-out cross-validation (LOOCV) method was used. Three ROC curves were plotted using the following features: (1) stage and age; (2) multiple biomarkers (gene expression levels); and (3) stage, age, and multiple biomarkers. The area under the curve (AUC) was calculated for each curve.

### 4.6. Pathway Analysis

Pathway analysis was performed using CancerCompass web-based tool (https://cancercompass.newgenes.org/, accessed on 6 January 2024) to assess the enrichment of 28 potential RNA biomarkers in cancer-related pathways. Cancer-related genes were collected from multiple cohorts, and consensus cancer genes were defined as those present in more than two databases. Cancer-related pathways were identified using a hypergeometric test. In the process of pathway analysis, a cutoff of false discovery rate control was set to 0.01, and after removing duplicate pathways among the top 10 pathways satisfying *p*-value < 0.01, cancer-relevant pathways were selected. A Sankey plot was provided by CancerCompass and a waterfall plot was generated using R software 4.3.0.

### 4.7. Statistical Analysis and Visualization

R software (version 4.3.0, R Foundation for Statistical Computing, Vienna, Austria) was used for the study. The Cox regression analysis was performed using the survival packages. The Kaplan–Meier plot and log-rank test were performed using the survminer (https://cran.r-project.org/web/packages/survminer/index.html, accessed on 6 January 2024) package. The randomForest R package (https://cran.r-project.org/web/packages/randomForest/, accessed on 6 January 2024) was used for the random forest machine learning analysis. The stats R package was employed for fitting the logistic regression model (https://www.R-project.org, accessed on 6 January 2024). The porch R package was utilized for generating the ROC curve and calculating AUC values [51]. Finally, the ComplexHeatmap 2.17.0 package was used for the heatmap analysis.

## 5. Conclusions

Through Cox regression analysis, which considers multiple variables across multiple cohorts, both novel and known prognostic biomarkers were identified. The results of the study will contribute to precision medicine research in determining patient prognosis in colorectal cancer in the future.

## Figures and Tables

**Table 1:** Clinical and histological characteristics of three cohorts.

Features		TCGA (n = 422)	GSE17536 (n = 177)	GSE39582 (n = 557)
		Case Number (Proportion)		
Age (Mean and SD)		66.4 ± 12.9	65.5 ± 13.1	66.8 ± 13.3
Gender	Male	256 (53.6%)	96 (54.2%)	305 (54.8%)
	Female	196 (46.4%)	81 (45.8%)	252 (45.2%)
Stage	I	73 (17.3%)	24 (13.6%)	31 (5.6%)
	II	165 (39.1%)	57 (32.2%)	262 (47.0%)
	III	123 (29.1%)	57 (32.2%)	204 (36.6%)
	IV	61 (14.5%)	39 (22.0%)	60 (10.8%)
MSI	Yes (or dMMR)	11 (2.6%)	N/A	71 (12.7%)
	No (or pMMR)	79 (18.7%)	N/A	440 (79.0%)
Histologic type	Adenocarcinoma	356 (84.4%)	N/A	N/A
	Mucinous adenocarcinoma	61 (14.5%)	N/A	N/A
	Other types	5 (1.1%)	N/A	N/A

N/A, not available.

**Table 2:** Results of the Cox regression analysis. After excluding genes that did not satisfy the Cox proportional hazard assumptions, 28 genes were selected based on the following criteria: (1) the direction of hazard ratios is the same in all three cohorts; and (2) the *p*-value of gene expression levels is less than 0.05 in all three cohorts. The hazard ratios (HRs) and *p*-values shown in the table are derived from a multiple Cox regression model with four variables: sex, age, stage, and gene expression level.

Gene Symbol (Probe Name)	TCGA-COAD (n = 422)		GSE17536 (n = 177)		GSE39582 (n = 557)	
	HR	*p*-Value	HR	*p*-Value	HR	*p*-Value
*PI4K2A* (209345_s_at)	1.766	0.03	9.269	0	1.571	0.022
*BAHD1* (203051_at)	1.936	0.011	4.052	0.005	1.67	0.031
*MFAP1* (203406_at)	1.84	0.022	2.561	0.006	1.407	0.049
*OAZ2* (201365_at)	1.829	0.041	2.392	0.026	1.51	0.043
*FAM219B* (224804_s_at)	1.815	0.011	2.348	0.016	1.51	0.019
*KCTD1* (226246_at)	1.3	0.004	2.583	0.009	1.303	0.014
*DNAJA4* (1554334_a_at)	1.6	0.049	2.069	0.002	1.297	0.024
*ZEB1-AS1* (229090_at)	1.383	0.018	1.839	0.015	1.463	0.001
*OSBPL1A* (209485_s_at)	1.262	0.036	1.723	0.006	1.238	0.007
*MYRIP* (214156_at)	0.901	0.016	0.69	0.002	0.866	0.017
*TOX3* (215108_x_at)	0.854	0.033	0.726	0.009	0.844	0.017
*F2RL2* (230147_at)	0.869	0.026	0.687	0.016	0.815	0.001
*FGFR4* (204579_at)	0.831	0.046	0.673	0.049	0.852	0.047
*KRTAP4-1* (234635_at)	0.937	0.043	0.547	0.009	0.762	0.025
*CHN2* (207486_x_at)	0.774	0.013	0.527	0.013	0.78	0.023
*NFE2L3* (236471_at)	0.7	0.041	0.625	0.004	0.754	0.001
*TTTY14* (207063_at)	0.899	0.013	0.384	0.046	0.774	0.05
*CACNA1D* (1555993_at)	0.738	0	0.518	0.037	0.776	0.021
*SLC18A1* (207074_s_at)	0.939	0.045	0.251	0.007	0.775	0.025
*CMC1* (228283_at)	0.644	0.028	0.519	0.035	0.706	0.012
*ITGB8-AS1* (230446_at)	0.8	0.021	0.416	0.002	0.638	0.001
*LARS2* (204016_at)	0.621	0.019	0.459	0.027	0.661	0.017
*RAB11FIP4* (225739_at)	0.681	0.042	0.466	0.003	0.576	0
*LINC00511* (230812_at)	0.798	0.043	0.302	0.036	0.611	0.015
*CHDH* (1559591_s_at)	0.739	0.01	0.314	0.004	0.648	0.008
*HNF1B* (208135_at)	0.842	0.037	0.053	0	0.598	0.008
*PDPK1* (221244_s_at)	0.469	0.001	0.181	0.012	0.582	0.027
*EIF1B* (237988_at)	0.602	0.017	0.025	0.006	0.333	0.015

## Data Availability

The RNA expression data and clinical data are available in the GEO under the accession number GSE17536 and GSE39582 and in the UCSC XENA website (https://xena.ucsc.edu/, accessed on 6 January 2024).

## Supplementary Materials

The supporting information can be downloaded at: https://www.mdpi.com/article/10.3390/ijms25063317/s1.
