# Supplementary material for: Unveiling Prognostic RNA Biomarkers through a Multi-Cohort Study in Colorectal Cancer
Source: Int J Mol Sci. 2024 Mar 14;25(6):3317. doi: 10.3390/ijms25063317 (PMC10969897; doi:10.3390/ijms25063317)
Supplement: Supplementary file 1 [file ijms-25-03317-s001.zip › Supplementary Figure S1.pdf]

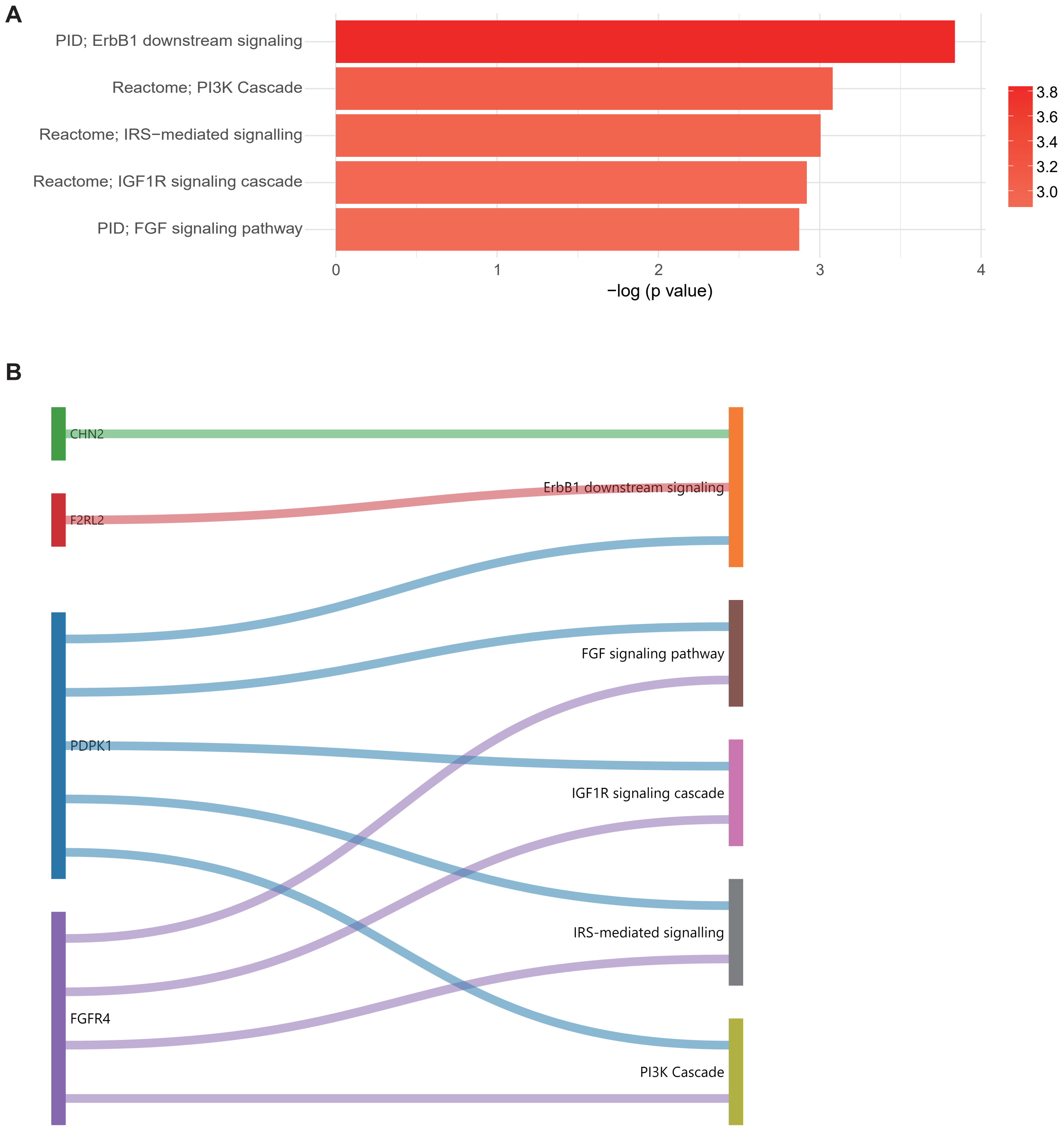

Figure S1: Enriched pathways and Sankey plot. (**A**) The waterfall plot of 5 pathways Pathway analysis results presented in a waterfall plot, indicating a total of 5 pathways. (**B**) Sankey plot of 5 pathways. The plot represents which gene were enriched in 5 pathways.
